# Recent insights on the impact of SWELL1 on metabolic syndromes

**DOI:** 10.3389/fphar.2025.1552176

**Published:** 2025-03-21

**Authors:** Mianhong Qin, Xuejie Yi, Ziqiang Duan, Bo Chang, Tao Li

**Affiliations:** ^1^ School of Sports Health, Shenyang Sport University, Shenyang, Liaoning, China; ^2^ College of Sport Science, Zhuhai College of Science and Technology, Zhuhai, Guangdong, China

**Keywords:** SWELL1, volume-regulated anion channel, metabolic syndrome, glucose metabolism, lipid metabolism

## Abstract

SWELL1 is a key component of the volume-regulated anion channel (VRAC) and participates in cell volume regulation as an ion channel plasma membrane protein. While early studies focused on its role in immune cell development and tumor progression, recent studies have revealed that SWELL1 plays an important role in metabolic diseases. Studies have shown that SWELL1 is extensively involved in physiological processes in peripheral metabolic tissues, including adipocyte hypertrophy, skeletal muscle volume regulation, insulin secretion, and hepatic lipid metabolism through interactions with the insulin signaling pathway. These functions play key roles in the pathogenesis of obesity, type 2 diabetes mellitus (T2DM), and non-alcoholic fatty liver disease (NAFLD), suggesting that SWELL1 may be a new target for the treatment of metabolic diseases. In this review, we focus on the structural and functional characteristics of SWELL1 to provide an in-depth explanation of its role in the development of metabolic syndrome, especially the regulation of the insulin signaling pathway, and provide a basis for the development of therapeutic strategies for metabolic diseases targeting SWELL1.

## 1 Introduction

The volume-regulated anion channel (VRAC) is an ion channel that is ubiquitous in animal cells and is primarily responsible for cell volume regulation under various stressors (Hoffmann et al., 2009; Hong et al., 2022). Since its discovery in 1980, identifying the molecular components of VRAC has been a central focus of research in this field, with the most recent and significant being the discovery that leucine-rich repeat-containing protein 8A (LRRC8A, also known as SWELL1) is an essential component of VRAC (Qiu et al., 2014; Voss et al., 2014). This discovery elucidated the molecular identity of VRAC and provided crucial insights into its structure and function, thereby considerably advancing progress (Mindell, 2014).

Structurally, SWELL1 has a unique N-terminal pore structural domain and a C-terminal leucine-rich repeat sequence, which can form a heterohexamer with other LRRC8 family members to regulate substrate specificity and VRAC functionality (Yamada and Strange, 2018; Zhou et al., 2018; Nakamura et al., 2020). SWELL1 is widely expressed in various tissues, including adipose tissue, skeletal muscle, liver, pancreatic islets, brain, and kidney (Kubota et al., 2004; Qiu et al., 2014; Zhang et al., 2017; Kang et al., 2018; Kumar et al., 2020). In addition to being a key component of VRAC involved in cell volume regulation, SWELL1 functions as a multifunctional regulatory molecule, playing a significant role in essential biological processes such as glucose and lipid metabolism, apoptosis, cell proliferation and differentiation, and insulin secretion (Zhang et al., 2017; Kang et al., 2018; Kofuji and Araque, 2019; Lu et al., 2019; Kumar et al., 2020; Wang et al., 2022). These findings provide new perspectives for in-depth investigations into the link between cell volume and metabolic regulation.

Emerging research underscores the critical role of SWELL1 in metabolic syndromes. Dysregulated SWELL1 expression in key metabolic tissues—such as adipose tissue, skeletal muscle, pancreas, and liver—has been shown to disrupt insulin signaling, impair insulin secretion, and alter hepatic lipid metabolism (Zhang et al., 2017; Kumar et al., 2020; Gunasekar et al., 2023). These dysfunctions contribute to the development of metabolic syndromes, including obesity, type 2 diabetes mellitus (T2DM), and non-alcoholic fatty liver disease [NAFLD] (Xie et al., 2017; Gunasekar et al., 2022; Gunasekar et al., 2023).

Although the structural features and metabolic regulatory functions of SWELL1 have been preliminarily characterized, the specific molecular mechanisms underlying its role in metabolic regulation remain to be fully elucidated (Kang et al., 2018). This review aims to provide a comprehensive analysis of the structural features and biological functions of SWELL1, with a particular focus on its role and potential mechanisms in metabolic syndromes. It highlights the role of SWELL1 in adipocytes, skeletal muscle cells, pancreatic β-cells, and hepatocytes, as well as its regulatory mechanisms in glucolipid metabolism. By synthesizing recent findings, this review seeks to advance the understanding of metabolism-related disease pathogenesis and support the development of novel therapeutic strategies.

## 2 Discovery, function, and structural features of SWELL1

### 2.1 Discovery and functional study of SWELL1

SWELL1 originally identified as LRRC8A (leucine-rich repeat-containing 8A). In 2003, Sawada et al. discovered the LRRC8A gene at the breakpoint of a chromosome 9 translocation in a patient with agammaglobulinemia, a congenital immunodeficiency syndrome. This chromosomal translocation led to the expression of both intact and truncated LRRC8A proteins in the patient. To investigate the effects of the LRRC8A mutation, the researchers introduced the mutant LRRC8A into the patient’s CD34^+^ bone marrow progenitor cells using retroviral vectors and transplanted these transduced cells into radiation-treated recipient mice to reconstitute the hematopoietic system. Their results demonstrated that the truncated LRRC8A protein significantly inhibited the differentiation of pro-B cells into pre-B cells, highlighting the critical role of this gene in B cell development (Sawada et al., 2003; Kubota et al., 2004).

This discovery spurred early studies on LRRC8A, particularly its role in immune system functions (Voss et al., 2014). A major breakthrough occurred in 2014 when two independent research teams confirmed LRRC8A as a key component of VRAC (Qiu et al., 2014; Voss et al., 2014). Qiu et al. established that LRRC8A is an essential VRAC component by combining a fluorescent cytometric assay (hypotonic-induced YFP quenching assay) with a genome-wide RNA interference (RNAi) screen. Based on their findings, LRRC8A was renamed SWELL1, with “SWELL” signifying its central role in regulating cellular swelling (Qiu et al., 2014). Similarly, Voss et al. employed a genome-wide small interfering RNA (siRNA) screen, along with zinc finger nuclease and CRISPR-Cas gene-editing technologies, to demonstrate that SWELL1 deletion significantly reduces VRAC currents, impairing the ability of cells to adapt to osmotic pressure changes. These pivotal findings provided the foundation for subsequent in-depth studies of the structural and functional characteristics of SWELL1 ion channels (Kefauver et al., 2018; Wu et al., 2023).

#### 2.1.1 SWELL1 mediates the VRAC regulation of cell volume

SWELL1, a core protein of VRAC, plays a key role in maintaining cell volume homeostasis (Wang et al., 2017; Centeio et al., 2020; Carpanese et al., 2024). When cells are in a hypotonic environment, the rapid influx of water molecules leads to cell swelling (Baumann et al., 2024). Consequently, SWELL1-mediated VRACs are rapidly activated to promote chloride ion and osmotic solute efflux, thereby regulating cell volume (Hoffmann et al., 2009; Syeda et al., 2016; Deneka et al., 2018; Liu et al., 2019; Centeio et al., 2020; Serra et al., 2021). Electrophysiological studies have provided key evidence to elucidate SWELL1 function. A characteristic outward chloride current, I_Cl (swell)_, is detected during cell swelling and is widely used as a key indicator for assessing VRAC activity (Liu et al., 2019; Brignone et al., 2022). Patch-clamp studies have shown that SWELL1 knockdown almost completely eliminates I_Cl (swell)_ induced by cell swelling (Qiu et al., 2014; Voss et al., 2014). This finding confirms that SWELL1 is essential for the normal function of VRAC, highlighting its critical role in cell volume regulation.

Although the critical role of SWELL1–VRAC in cell volume regulation has been well validated (Qiu et al., 2014; Voss et al., 2014; Wang et al., 2017; Centeio et al., 2020; Carpanese et al., 2024), the molecular mechanisms underlying the involvement of SWELL1 in volume sensing and its regulatory features in different cell types remain unclear. Exploring these issues in-depth will help to comprehensively elucidate the molecular mechanisms of cell volume regulation and open new possibilities for the treatment of related diseases.

#### 2.1.2 SWELL1 regulates T cell development

T cells are a core component of the adaptive immune system, and their development is regulated by the thymic microenvironment (Han and Zúñiga-Pflücker, 2021; Lin et al., 2024). Mice with a global *SWELL1* knockout (*Lrrc8a−/−*) display severe thymic developmental defects, including markedly reduced thymic volume and cellularity (Kumar et al., 2014). In particular, the number of thymocytes significantly decreases from the double-negative (DN2) stage onwards (Kumar et al., 2014; Zhang et al., 2021). These developmental defects ultimately lead to a significant decrease in the number of peripheral T cells, impaired T cell proliferation, and a reduced proportion of effector memory T cells in *Lrrc8a* ^−/−^ mice (Kumar et al., 2014), suggesting that SWELL1 plays a crucial role in early T cell development.

The T cell receptor (TCR) signaling pathway is a key molecular pathway involved in T cell activation and differentiation. TCR is responsible for transducing extracellular antigen signals into cells, thereby regulating T cell immune responses (Stephen et al., 2009). In an OT-I CD8^+^ T cell model study, *SWELL1* deficiency was found to cause significant abnormalities in the TCR signaling pathway, manifested as decreased expression of the upstream molecule Nur77 and weakened phosphorylation of downstream signaling molecules S6 and MAPK [ERK1/2] (Wang et al., 2023). The abnormal expression of these molecules leads to decreased T cell proliferation, reduced secretion of effector factors (TNFα and IFNγ), and downregulated expression of nutritional metabolism-related molecules [CD71 and CD98] (Wang et al., 2023). It has been reported that the TCR signaling pathway mainly regulates the biological behavior of T cells by activating the PI3K/AKT signaling pathway (Pompura and Dominguez-Villar, 2018). In wild-type thymocytes, cross-linking mediated by anti-SWELL1 monoclonal antibodies can significantly induce AKT phosphorylation, whereas PI3K inhibitors can completely block this phosphorylation reaction (Kumar et al., 2014), indicating that PI3K plays a critical role in SWELL1-mediated signaling. Another study reported that during T cell activation, TCR binds to antigens and transmits signals through CD3 molecules, thereby activating lymphocyte specific protein tyrosine kinase [LCK] (Liu Q. et al., 2024). Activated LCK promotes TCR-related ITAM motif phosphorylation, providing binding sites for Zeta chain-associated protein kinase 70 (ZAP70) and activating it (Suzuki et al., 2012). Immunoprecipitation experiments revealed that SWELL1 forms stable complexes with LCK the GRB2–GAB2 complex [growth factor receptor-bound protein 2 and GRB2-associated binding protein 2] (Kumar et al., 2014). In addition, SWELL1-mediated AKT phosphorylation is significantly reduced in *ZAP70* ^−/−^ thymocytes (Kumar et al., 2014). These results indicate that SWELL1 activates AKT through the LCK-ZAP-70-GAB2-PI3K pathway, thereby regulating T cell function. To eliminate the influence of other cellular factors, researchers also constructed a *LRRC8A* ^*−/−*^ *→Rag2* ^*−/−*^ chimeric mouse model. In this model, not only were the phenotypic characteristics of *Lrrc8a* ^*−/−*^ mice reproduced, but their thymocytes also showed significant proliferation disorders under anti-CD3 antibody and IL-2 stimulation (Kumar et al., 2014), further confirming the direct involvement of SWELL1 in T cell developmental regulation.

In summary, SWELL1 regulates T cell development by integrating the TCR and AKT signaling pathways. The elucidation of this mechanism not only deepens the understanding of the regulation of T cell development but also provides a new theoretical basis and potential targets for the development of therapeutic strategies against immune system diseases.

### 2.2 Structural features of SWELL1

SWELL1 belongs to the LRRC8 family and contains five members (LRRC8A–E). As a core component, SWELL1 can be combined with the four other members in different ratios to form functionally diverse VRAC channel variants that regulate the transmembrane transport of different substances (Abascal and Zardoya, 2012; Syeda et al., 2016; Lutter et al., 2017; Kern et al., 2023).

**FIGURE 1:**
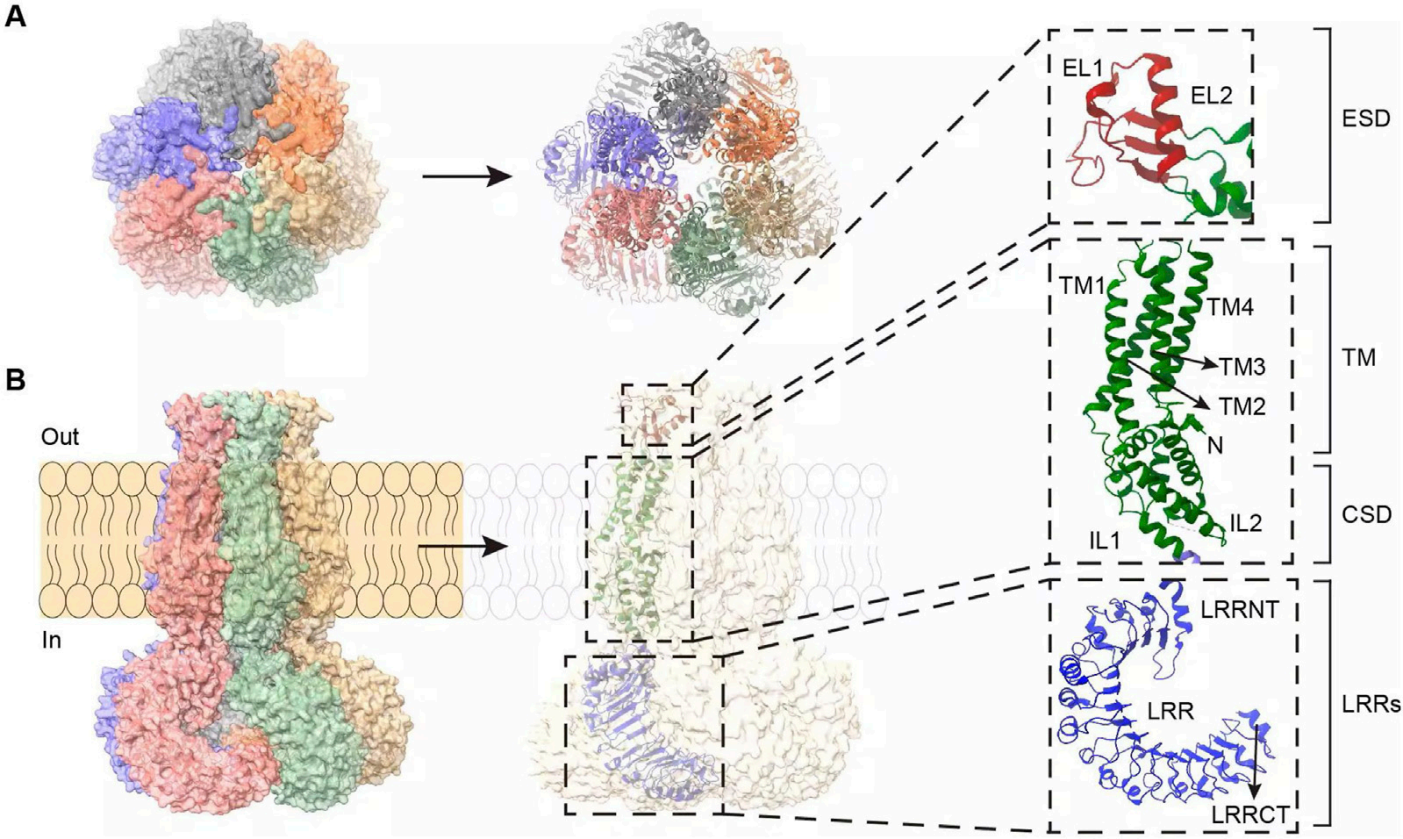
The overall structure and interaction sites of the SWELL1-LRRC8 subunit hexamer. **(A)** Bottom view of the overall structure of the SWELL1-LRRC8 subunit hexamer (observed from inside to outside the cell membrane). **(B)** Side view of the overall structure of the SWELL1-LRRC8 subunit hexamer (left, parallel to the cell membrane). SWELL1 sequence interaction site (right). N-terminus: ESD is the extracellular region composed of two extracellular loops, EL1 and EL2 (red); TM is a transmembrane region composed of TM1, TM2, TM3, and TM4 (green), with TM1 connecting ESD and TM to EL1; CSD is an intracellular region composed of IL1 and IL2 (green), TM2, TM3, and IL1 are connected to TM and CSD; LRRs are C-terminal leucine rich repeat sequences composed of LRRNT, LRR, and LRRCT (blue), connected by IL2 to TM4 and LRRs. LRRNT to LRRCT represent the alpha helices.

The *SWELL1* gene is highly conserved between humans and mice. It encodes a protein containing 810 amino acid residues with a molecular weight of approximately 95 kDa (Sawada et al., 2003; Kubota et al., 2004; Osei-Owusu et al., 2018). Structural biology studies have shown that SWELL1 consists of two major functional domains: N-terminal transmembrane structural domains (TMDs) and C-terminal leucine-rich repeat sequences [LRRs] (Kasuya et al., 2018; Osei-Owusu et al., 2018; Zhou et al., 2018) (Figure 1). TMDs constitute the core pore region of ion channels, including the extracellular region (ESD), transmembrane region (TM), and intracellular region [CSD] (Kasuya et al., 2018) (Figure 1). The ESD consists of two extracellular loops (EL1 and EL2) (Figure 1), which act as the narrowest part of the ion conduction pathway and are responsible for ion filtration and selection (Deneka et al., 2018; Liu H. et al., 2023). Heterodimers formed by SWELL1 and different LRRC8 members differ in pore size and polarity in this region, thereby regulating channel permeability in different matrices (Kasuya and Nureki, 2022). The TM region consists of four transmembrane helices (TM1–4) that connect the ESD to the CSD (Kasuya et al., 2018), forming the channel’s exit and connecting to the LRRs (Deneka et al., 2018) (Figure 1).

The LRRs, the second major functional domain, consist of three components—LRRNT, 15 LRRs, and LRRCT—arranged in a horseshoe-shaped structure (Figure 1). This domain is critical for protein–protein interactions and signal transduction (Kubota et al., 2004; Abascal and Zardoya, 2012). The structural integrity of LRRs is critical for SWELL1 activity, and their mutation or deletion leads to severely impaired VRAC function (Zhang et al., 2017). In the VRAC hexameric structure, the region of LRRs differs between different LRRC8 subunits, and this difference is reflected in the contact interface between the α-subunit (SWELL1) and the β-subunit (LRRC8B–E). Cryo-electron microscopy studies have shown that the LRR regions of neighboring subunits can form two distinct contact patterns: “tight” or “loose” (Kasuya and Nureki, 2022). When the α and β subunits are adjacent one another, the LRRs region is tightly bound; conversely, the binding is loose when the β and γ subunits are adjacent one another. While the physiological significance of this structural feature is unclear, it may be related to channel assembly and regulation. However, further experimental evidence is needed to verify this hypothesis.

Currently, structural biology research on SWELL1 is mainly based on chimeric SWELL1 channels [LRRC8C-LRRC8A (IL1–25] (Takahashi et al., 2023). While these chimeric constructs replicate some functional properties of natural VRACs (Yamada et al., 2021), they fail to fully capture the physiological state of VRACs in mammalian cells due to the absence of essential post-translational modifications. Resolving the structure of SWELL1 *in situ* within mammalian cells remains crucial for a deeper understanding of the molecular mechanisms underpinning VRAC functionality and its roles in pathophysiological processes.

## 3 SWELL1 and metabolic syndrome

### 3.1 SWELL1 and obesity

Obesity is a complex chronic metabolic disease, the mechanism of which involves functional abnormalities across multiple tissue systems. SWELL1, an emerging metabolic regulator, plays a key role in adipose tissue and skeletal muscle, contributing to obesity development by modulating energy metabolism and fat accumulation.

#### 3.1.1 SWELL1 regulates adipocyte hypertrophy

Adipocyte hypertrophy is an important feature in the development of obesity (Muir et al., 2016; Chen et al., 2020; Chen et al., 2022). Several studies have confirmed the close relationship between SWELL1 and adipocyte hypertrophy. In a high-fat diet (HFD)-induced obese mouse model, SWELL1 was significantly upregulated in inguinal white adipose tissue (iWAT) and epididymal white adipose tissue (eWAT), and its expression was significantly positively correlated with adipocyte volume (Xie et al., 2017). A patch-clamp study confirmed that the current density mediated by SWELL1 was significantly increased in adipocytes of obese mice (Zhang et al., 2017). This finding was also validated by a clinical study in which visceral adipocyte volume and SWELL1-mediated currents were significantly higher in patients deemed obese (BMI >30) than in patients that were not deemed obese [BMI <30] (Zhang et al., 2017). qPCR analysis of adipocytes from obese mice showed that the mRNA expression of SWELL1 and its associated LRRC8 subunits (LRRC8b and LRRC8d) were upregulated (Zhang et al., 2017; Sawicka and Dutzler, 2022). Considering that SWELL1 is an essential component of VRAC, which plays a key role in cell volume regulation (Menegaz et al., 2019), these results suggest that the SWELL1–VRAC complex may be synergistically involved in the adaptive regulation of adipocyte cell volume hypertrophy through an undefined mechanism.

To verify the role of SWELL1 in adipocyte volume regulation, Zhang et al. adopted a systematic research strategy, progressing from short-term to long-term studies and local to systemic analyses. They first performed a 12-day short-term knockdown of *SWELL1* in the iWAT of obese mouse using adenovirus-mediated RNAi (shSWELL1-mCherry) and found a 28% reduction in the total adipocyte volume. Long-term knockdown using the AAV/Rec2-shSWELL1-mCherry vector for 16 weeks maintained the reduction in adipocyte volume and significantly decreased systemic fat content, particularly visceral fat (e.g., eWAT), while mildly increasing lean body mass (Zhang et al., 2017). These results preliminarily confirmed the causal relationship between SWELL1 expression and adipocyte volume regulation. To gain a deeper understanding of the physiological functions of SWELL1 in adipose tissue, Zhang et al. also constructed adipose tissue-specific *SWELL1* knockout (Adipo KO) mice (Zhang et al., 2017). Unlike RNAi methods, this genetic approach eliminates off-target effects and provides insights into the role of SWELL1 in adipose tissue development. While Adipo KO mice exhibited normal phenotypes on a regular diet, they displayed distinct metabolic traits under HFD conditions. These included slower weight gain, reduced adipose tissue mass, and increased lean body mass ratios. However, adverse metabolic outcomes also emerged, such as impaired glucose tolerance and reduced insulin sensitivity (Zhang et al., 2017). This phenotypic duality suggests that SWELL1 has complex regulatory functions in adipose tissue, not only participating in adipocyte volume regulation but also possibly affecting systemic metabolic homeostasis through unknown mechanisms. These findings provide a new direction for further research into the functional mechanisms of SWELL1.

#### 3.1.2 Role and molecular mechanisms of SWELL1 in regulating adipocyte metabolism

Obesity leads to an enlarged fat cell volume and is often accompanied by metabolic disorders of fat cells. To investigate the role of SWELL1 in the regulation of adipocyte metabolism, Zhang et al. (2017) used CRISPR/Cas9 gene-editing technology to construct a *SWELL1* knockout 3T3-F442A adipocyte line and a conditional knockout mouse model [*SWELL1* ^*fl*^] (Zhang et al., 2017). RNA sequencing and gene set enrichment analysis showed that *SWELL1* deletion led to significant changes in the expression of 9,090 genes, with the most significant changes observed in genes related to adipogenesis, glucose metabolism, and insulin signaling pathways (Zhang et al., 2017). Based on these transcriptomic findings, an in-depth metabolic phenotype analysis showed that, under high glucose conditions (25 mM), the lipid content in adipocytes lacking *SWELL1* was significantly reduced, whereas no significant changes were observed under low glucose conditions [5 mM] (Zhang et al., 2017). Radioactive tracing and electron microscopy analyses confirmed that *SWELL1* deficiency significantly inhibited glucose uptake under insulin stimulation, leading to a decrease in intracellular glycogen granules (Zhang et al., 2017). As glucose is a precursor of lipid synthesis (Jin et al., 2023), these results suggest that the glucose uptake disorder caused by *SWELL1* deficiency may be the direct cause of the decrease in lipid content under high glucose conditions.

**FIGURE 2:**
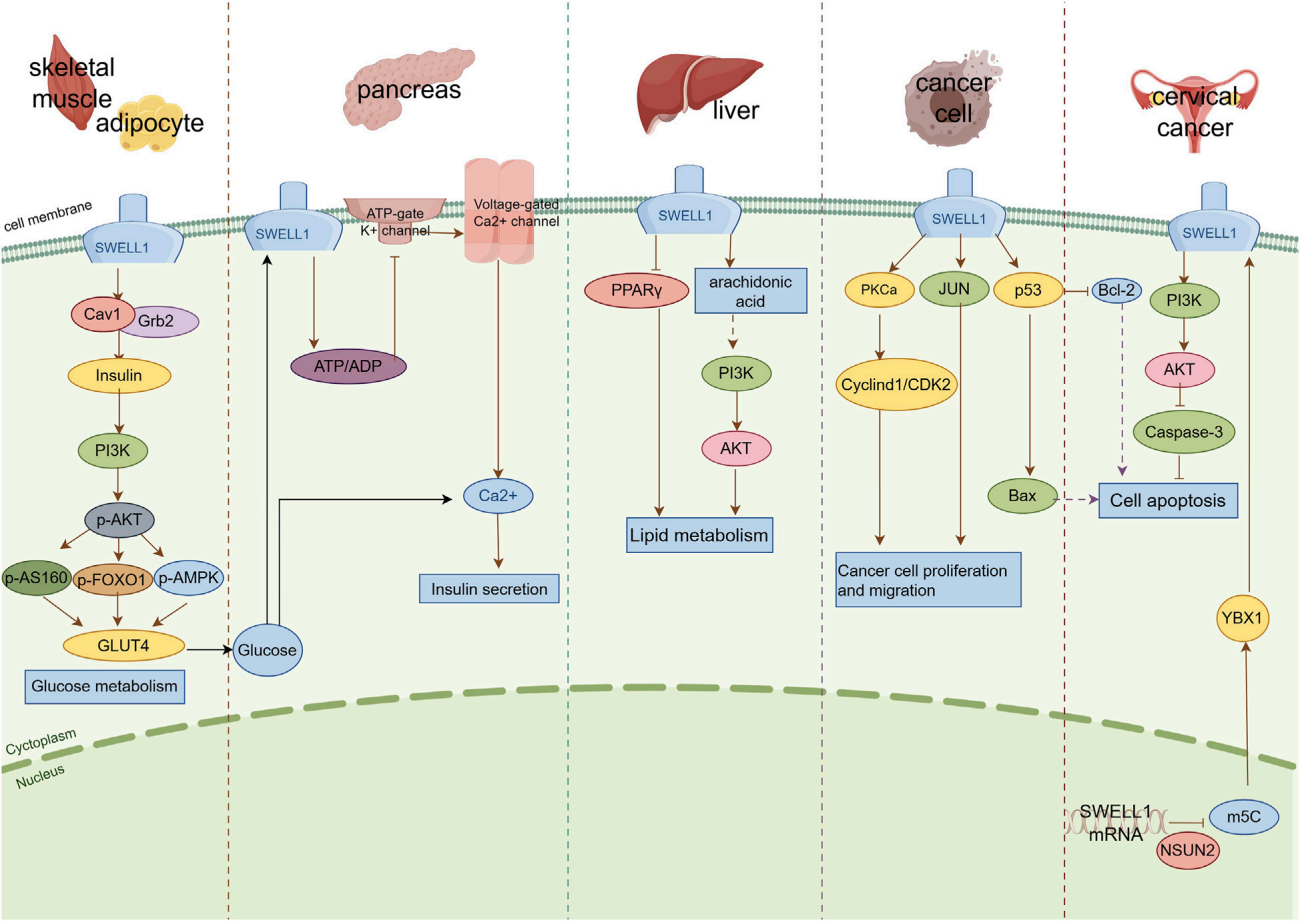
Schematic diagram of the signal transduction mechanism of SWELL1 in different tissues and cells. SWELL1 exerts regulatory effects in different tissues and cells through various signaling pathways: (1) Skeletal muscle and adipose tissue: SWELL1 forms a complex with Cav1 and Grb2 to regulate the insulin signaling pathway, thereby affecting glucose metabolism. (2) Pancreatic beta cells: Glucose metabolism or changes in cell volume activate SWELL1, which interacts with voltage-gated calcium channels to promote calcium influx and trigger insulin secretion. (3) Liver tissue: SWELL1 affects lipid metabolism by regulating PPAR γ signaling and arachidonic acid metabolism pathways. (4) Cancer cells: SWELL1 regulates cancer cell proliferation and migration through signaling pathways such as PKCa and JUN, and regulates cancer cell apoptosis through signaling pathways such as p53 and PI3K/AKT. The solid pointed arrow represents promotion, the solid flat arrow represents inhibition, and the dashed arrow represents potential or indirect mechanisms.

In this context, the insulin-PI3K-AKT signaling pathway is of particular importance, as it serves as the core pathway by regulating glucose metabolism. AKT2 is a key effector molecule that mediates the glucose transport regulated by insulin in this pathway (Beg et al., 2017). It is worth noting that regulation of the insulin signaling pathway not only depends on AKT2 but is also closely associated with other crucial molecules such as SWELL1. Research has demonstrated that SWELL1 forms functional complexes with caveolin-1 (Cav1) and GRB2 through its C-terminal LRR domain (Zhang et al., 2017; Alghanem et al., 2021). In this complex, Cav1 forms specialized microstructural regions (caveolas) on the cell membrane, providing a spatial basis for effective aggregation and interaction of signaling molecules (Rezola et al., 2021). GRB2, as a negative regulator of the insulin signaling pathway, can bind to insulin receptor substrates (IRS) and affect the transmission of insulin signals (Li and Spalding, 2022). In SWELL1 knockout adipocytes, the phosphorylation level of the AKT2 Ser474 site decreases after insulin stimulation, while the phosphorylation level of the AKT1 Ser473 site shows an abnormal increase (Zhang et al., 2017). Reducing *GRB2* expression through siRNA can significantly improve the damaged insulin-PI3K-AKT2 signaling pathway in *SWELL1* knockout adipocytes (Zhang et al., 2017). *In vivo* experiments have also shown that after reducing the expression of *SWELL1* in mouse iWAT tissues, insulin-induced AKT2 phosphorylation significantly decreased, whereas AKT1 phosphorylation levels remained unchanged (Zhang et al., 2017). *SWELL1* deficiency also leads to the dysfunction of multiple effector molecules downstream of the insulin signaling pathway, including decreased phosphorylation levels of AS160, impaired transport of GLUT4 to the cell membrane, reduced glucose uptake, and significant changes in GSK3 β and transcription factor FOXO1 activity (Zhang et al., 2017) (Figure 2). These results reveal a possible molecular mechanism: in adipocytes, the absence of *SWELL1* enhances the activity of GRB2, leading to an increase in its binding to IRS, thereby inhibiting IRS activation of PI3K. This subsequently affects the phosphorylation level and activity of AKT2, ultimately leading to inhibition of insulin-mediated metabolic processes such as GLUT4 membrane translocation, glucose uptake, and lipid synthesis.

Taken together, SWELL1 is an important molecule in the insulin signaling pathway that regulates AKT2-mediated metabolic processes by forming protein complexes in specific regions of the cell membrane. This highly specific regulatory mechanism enhances our understanding of adipose tissue metabolism and offers new therapeutic targets for metabolic disease treatment.

#### 3.1.3 Role and molecular mechanisms of SWELL1 in regulating skeletal muscle metabolism

The integrity of skeletal muscle morphology and function depends on the precise regulation of skeletal muscle differentiation, which is crucial for maintaining homeostasis and energy metabolism (Kamei et al., 2020). In 2020, Kumar et al. explored the role of SWELL1 in skeletal muscle development using CRISPR/Cas9 gene-editing technology (Kumar et al., 2020). Following *SWELL1* knockout in C2C12 and primary skeletal muscle cells, myotube areas were reduced by 58% and 45%, respectively, and the myoblast fusion capacity was significantly decreased. Transcriptome analysis showed that *SWELL1* deficiency led to the downregulation of key genes (*Mef2a* and *Myl2*) in skeletal muscle differentiation, while pro-differentiation factors PGC1α and PPARγ showed modern compensatory upregulation (Krämer et al., 2006; Kumar et al., 2020). By constructing a skeletal muscle-targeted *Lrrc8a* KO (Skm KO) mouse model, histological analysis showed a 27% reduction in the tibial muscle cross-sectional area (Kumar et al., 2020), confirming the *in vitro* experimental results and the critical role of SWELL1 in muscle fiber development. In addition to affecting skeletal muscle morphology and development, SWELL1 deficiency causes significant metabolic changes. Skm KO mice maintained normal glucose tolerance and insulin sensitivity on a regular diet; however, their body fat increased by 29%, indicating relative obesity (Kumar et al., 2020). After being fed an HFD, these mice showed decreased glucose tolerance and mild insulin resistance (Kumar et al., 2020), indicating that SWELL1 has a protective effect against nutritional overload.

The insulin signaling pathway is an important regulator of skeletal muscle differentiation (Dadon-Freiberg et al., 2020). Western blotting analysis results showed that the phosphorylation levels of key molecules in the insulin signaling pathway, namely, pAKT2, pAS160, and pFOXO1, are significantly reduced in muscle tubes with *SWELL1* deficiency. Simultaneously, the transcription of downstream genes such as *GLUT4* and *FOXO3/4/6* is inhibited (Kumar et al., 2020) (Figure 2). In addition to insulin signaling, mechanical stimulation is an important factor in regulating skeletal muscle growth and development (Liu et al., 2011). Through mechanical stretching experiments, it was found that C2C12 myotube *SWELL1* deficiency significantly weakened the activation of the PI3K-AKT2/AKT1-pAS160-MAPK (ERK1/2) signaling pathway induced by mechanical stimulation (Kumar et al., 2020). This finding suggests that SWELL1 may serve as a key molecule that integrates mechanical stimulation and insulin signaling pathways to jointly regulate skeletal muscle development and metabolic adaptation. This dual regulation explains the simultaneous effects of SWELL1 deficiency on skeletal muscle morphology and metabolic function.

It is worth noting that SWELL1 exhibits different metabolic regulatory functions in skeletal muscle and adipose tissue (Zhang et al., 2017; Kumar et al., 2020). Adipo KO mice exhibit glucose intolerance and insulin resistance at baseline, and a HFD further exacerbates these metabolic disorders, but with less weight gain [10]. Under a normal diet, Skm KO mice mainly exhibit an increase in body fat, and metabolic abnormalities occur only under HFD stimulation (Kumar et al., 2020). This tissue-specific phenotypic difference suggests an interesting metabolic regulatory network: skeletal muscle SWELL1 deficiency may trigger adipose tissue remodeling through a compensatory mechanism, thereby maintaining systemic energy balance. This cross-tissue metabolic regulation broadens our understanding of SWELL1 function and provides a new framework for exploring metabolic communication between organs.

### 3.2 SWELL1 and T2DM

T2DM is a chronic metabolic disease characterized by high blood sugar levels, and its pathogenesis involves multiple aspects, such as decreased insulin secretion and peripheral tissue insulin resistance (Sun et al., 2023). In 2022, Gunasekar et al. found that the expression of SWELL1 protein in the pancreatic islets and white adipose tissue of T2DM mouse models was reduced by 66% and 38%, respectively. Similarly, the level of SWELL1 protein in the pancreatic islets and adipose tissue of postmortem specimens of patients with T2DM was approximately 50% lower than that in the non-diabetic control group (Gunasekar et al., 2022). Further research has shown that the I_Cl (swell)_ current density mediated by SWELL1 is significantly reduced in T2DM mice and human pancreatic β-cells, indicating impaired functional SWELL1 ion channel activity on the cell membrane. This abnormal ion channel function may ultimately lead to insulin secretion dysfunction by affecting cell membrane potential regulation and ion balance (Ye et al., 2022). These findings suggest that decreased expression and function of SWELL1 play important roles in the pathogenesis of T2DM.

Based on the key role of SWELL1 in T2DM, Gunasekar et al. developed a SWELL1 channel small-molecule modulator called SN-401. *In vitro* experiments showed that SN-401 effectively increased the expression of SWELL1 protein and stabilized the SWELL1–LRRC8 complex within a specific concentration range (100–500 nM), exhibiting dose-dependent effects (Gunasekar et al., 2022). In animal experiments, the active homolog SN-40X significantly improved systemic insulin sensitivity and glucose homeostasis in T2DM mice without significant toxicity or risk of hypoglycemia. Importantly, SN-40X did not affect glucose metabolism in non-diabetic mice, underscoring its disease-specific action and strong potential as an ideal therapeutic drug.

#### 3.2.1 Role and molecular mechanisms of SWELL1 in regulating β-cell function

The insulin secretion dysfunction of pancreatic β-cells plays a key role in the pathogenesis of T2DM. In insulinoma (MIN6) cells and mouse and human primary β-cells, SWELL1 mediates the depolarization currents of β-cell membranes induced by hypotonic or glucose stimulation (Kang et al., 2018) (Figure 2). When *SWELL1* is absent, the response of β-cells to glucose stimulation is weakened, manifesting as a transient decrease in Ca^2+^ levels and insulin secretion (Kang et al., 2018). This phenomenon has been further validated in animal models: β-cell specific *SWELL1* knockout mice not only exhibit reduced insulin secretion stimulated by glucose, but also exhibit decreased glucose tolerance (Kang et al., 2018). In the context of mild obesity, these mice exhibit severe metabolic disorders, including elevated fasting blood glucose levels and impaired glucose tolerance (Kang et al., 2018). These findings indicate that SWELL1 plays an important regulatory role in the insulin secretion function of β-cells, and its absence can lead to β-cell dysfunction, thereby affecting glucose metabolism homeostasis.

Interestingly, Stuhlmann et al. (2018) provided a new perspective. They found that the absence of *SWELL1* did not completely block the response of β-cells to glucose, but delayed processes such as cell excitation, Ca^2+^ elevation, and insulin secretion. This finding underscores the complexity of insulin secretion regulation. Moreover, in addition to SWELL1, ATP-sensitive K^+^ channels (KATP channels) and voltage-gated calcium channels are also involved in regulating insulin secretion (Cornell et al., 2022; Lorza-Gil et al., 2023). These ion channels may have functional interactions or synergistic effects with SWELL1, jointly forming a precise regulatory network (Best et al., 2011; Stuhlmann et al., 2018) (Figure 2).

In summary, SWELL1 plays a crucial role in glucose-stimulated insulin secretion by regulating β-cell membrane potential and ion channel function (Kang et al., 2018; Stuhlmann et al., 2018). Exploring the interactions between SWELL1 and other regulatory factors can enhance understanding of β-cell function and provide valuable directions for developing novel T2DM treatment strategies.

### 3.3 SWELL1 and NAFLD

NAFLD is a chronic liver disease characterized by abnormal lipid deposition in liver cells (Ogurtsova et al., 2017; Ipsen et al., 2018; Younossi et al., 2018), and its pathogenesis is closely related to adipose tissue function. Under normal conditions, adipose tissue, the main energy storage organ in the body, can cope with overnutrition through adaptive expansion. When the adaptive expansion function is impaired, compensatory lipolysis occurs in adipose tissue, leading to the release of large amounts of free fatty acids into the bloodstream and their deposition in the liver (Lonardo et al., 2017). Recent studies have shown that SWELL1 plays a key regulatory role in this process.

In 2023, Gunasekar et al. found that Adipo KO mice exhibited restricted adipose tissue expansion under an HFD, accompanied by elevated levels of plasma non-esterified free fatty acids and glycerol. When these mice received high-fat, high-sugar, and gubra amyloid NASH dietary interventions, a series of typical NAFLD pathological changes were observed in the liver, including increased liver mass, hepatocyte hypertrophy, and exacerbation of large and small vesicular steatosis. Simultaneously, the content of diacylglycerols (DAGs) and triglycerides (TAGs) rich in oleic acid in the liver significantly increased, and the inflammatory response was enhanced (manifested as a 2-fold increase in macrophage marker CD68 expression and upregulation of PPARγ expression) (Gunasekar et al., 2023). These results indicate that the specific absence of SWELL1 in adipose tissue is closely related to restricted adipose tissue expansion, elevated plasma lipid levels, and enhanced hepatic lipid deposition and inflammatory responses.

It is worth noting that SWELL1 is of great significance in the study of pathogenesis and shows promising prospects for treatment. Notably, the SWELL1 small-molecule inducer SN-401 significantly improves the NAFLD phenotype of ob/ob and db/db diabetic mice, including reducing liver weight and triglyceride concentration and alleviating liver steatosis and cell damage (Gunasekar et al., 2022). This finding confirms that SWELL1 deficiency leads to the exacerbation of NAFLD, further supporting the key regulatory role of SWELL1 in the development of NAFLD.

Research has also highlighted a SWELL1-centered adipose-liver axis. SWELL1 deficiency in adipose tissue impairs lipid storage capacity, leading to elevated circulating free fatty acids. This, in turn, promotes hepatic lipid accumulation and inflammatory activation, ultimately accelerating NAFLD progression (Gunasekar et al., 2023). These findings not only deepen our understanding of NAFLD pathogenesis but also position SWELL1 as a novel therapeutic target for its treatment.

#### 3.3.1 Role and molecular mechanism of SWELL1 in hepatic lipid metabolism

The dysregulation of lipid metabolism is an important pathological mechanism that leads to lipid accumulation in the liver. Studies have found that in the hepatitis B virus (HBV)-positive liver cancer cell line HepG2.2.15, ethanol treatment not only leads to an increase in intracellular triglyceride and cholesterol levels but also synchronously upregulates the expression of HBV X protein/pregenomic RNA (HBx/pgRNA), HBV X protein (HBx), and SWELL1, which is not observed in HBV-negative HepG2 cells (Liu et al., 2020). Inhibition of *SWELL1* expression by siRNA significantly reduces lipid levels in HepG2.2.15 cells, indicating that SWELL1 plays an important role in ethanol- and HBV-co-induced lipid accumulation in liver cells. Additionally, mechanistic studies revealed that HBx can dose dependently promote the transcription and expression of SWELL1 (Liu et al., 2020). Further bioinformatics analysis combined with luciferase reporter gene and chromatin immunoprecipitation experiments revealed that the transcription factor Sp1 is a key mediator in this regulatory process, mediating the HBx transcriptional activation of SWELL1 by specifically binding to the core region of the SWELL1 promoter (−1,381/-781). This conclusion is further supported by *Sp1* knockdown experiments, which showed that inhibiting *Sp1* not only reduced the expression of SWELL1 but also decreased intracellular lipid accumulation (Liu et al., 2020). These findings establish the HBx-Sp1-SWELL1 pathway as a critical regulator of lipid accumulation in liver cells.

Arachidonic acid is an important polyunsaturated fatty acid (Wang et al., 2024) that can be converted into metabolites such as prostaglandin E2 (PGE2) and leukotriene D4 (LTD4) by various enzymes (Blitek and Szymanska, 2020). These metabolites can regulate processes such as liver fatty acid uptake and triglyceride synthesis (Nakamura et al., 2014). Liu et al. found a positive correlation between the expression level of SWELL1 and the production of PGE2 and LTD4 production in HBV transgenic mouse models and various liver cell lines (HepG2, HepG2.2.15, and K180) (Liu et al., 2020). *In vitro* experiments further confirmed that overexpression of *SWELL1* in HepG2 cells increased the levels of PGE2 and LTD4 in the culture medium, while knockdown of *SWELL1* inhibited this process (Liu et al., 2020). Moreover, clinical analyses revealed a strong correlation between SWELL1 expression and key enzyme genes involved in the arachidonic acid metabolism pathway, such as PGE2 synthase *PTGES2* and LTD4 synthase *GGT5* (Liu et al., 2020). These results suggest that SWELL1 affects liver lipid metabolism by regulating the arachidonic acid metabolism pathway (Figure 2).

Although the above studies have revealed the important role of SWELL1 in liver lipid metabolism, the detailed molecular mechanism requires further clarification. Previous studies have shown that SWELL1 can regulate the PI3K AKT signaling pathway in metabolic tissues such as fat and skeletal muscle (Zhang et al., 2017; Kumar et al., 2020). Interestingly, PGE2 can also activate this pathway through its receptors (Wang et al., 2015), suggesting that SWELL1 regulates liver lipid metabolism via a similar mechanism (Figure 2). Future investigations into the molecular mechanisms underlying SWELL1 function will deepen our understanding of liver lipid metabolism disorders and pave the way for developing novel SWELL1-targeted therapies.

Overall, current research has revealed the important regulatory role of SWELL1 in major metabolic tissues such as adipose tissue, skeletal muscle, and liver. These findings not only deepen people’s understanding of the physiological functions of SWELL1 but also provide new ideas and strategies for the prevention and treatment of metabolic diseases. However, existing research still has certain limitations. At present, genetic research on the function of SWELL1 is mainly limited to models of functional loss such as gene knockout or knockdown. Although these models provide valuable information, the loss of SWELL1 function may activate compensatory mechanisms and mask some of its physiological effects. In addition, the lack of a SWELL1 activity enhancement model makes it difficult to comprehensively evaluate its dose-response relationship with metabolic phenotypes. Future research should expand genetic manipulation methods, construct SWELL1 overexpression and functional acquisition mutation models, and thoroughly explore the effects of enhanced SWELL1 activity on adipose tissue remodeling, skeletal muscle development, and systemic metabolic homeostasis, revealing its potential association with the risk of metabolic diseases. Meanwhile, considering the functional differences exhibited by SWELL1 in different tissues, future research needs to further elucidate its tissue-specific mechanism of action and explore the synergistic regulatory network between different metabolic tissues.

## 4 Role of SWELL1 in other physiological and pathological processes

Although the role of SWELL1 in metabolic syndrome and metabolic disorders is receiving increasing attention, its functional effects go far beyond that. SWELL1 is also involved in regulating various other physiological and pathological processes, including the proliferation, migration, and apoptosis of cancer cells. These functions may be closely related to the multifunctionality of SWELL1 in cell volume regulation, ion homeostasis, and signal transduction. In fact, metabolic disorders (such as obesity and type 2 diabetes) are closely related to cancer. Research has shown that metabolic abnormalities such as insulin resistance and chronic inflammation may promote cancer cell proliferation and invasion by affecting cellular energy metabolism and signal transduction (Jiang et al., 2012). Considering the important role of SWELL1 in regulating insulin sensitivity and cell proliferation, it may play a crucial bridging role between metabolic disorders and cancer. Exploring the specific mechanisms of SWELL1 in these pathological processes can help elucidate the intrinsic relationship between metabolic abnormalities and cancer development, providing new ideas and strategies for metabolic tumor therapy.

### 4.1 SWELL1 regulates cancer cell proliferation and migration

Abnormal cell proliferation and migration are key features of cancer occurrence and development (Doran et al., 2024; Liu S. et al., 2024; Zhou et al., 2024). Multiple studies have revealed that SWELL1 is upregulated in various cancers and regulates cancer cell proliferation and migration through different signaling pathways (Rubino et al., 2018; Zhang et al., 2018; Konishi et al., 2019; Lu et al., 2019; Xu et al., 2022; Liu T. et al., 2023).

Clinical studies have shown that, compared to normal tissues, the expression levels of SWELL1 in pancreatic cancer (PAAD), hepatocellular carcinoma (HCC), esophageal cancer, colon cancer, and malignant glioma tissues are significantly increased (Rubino et al., 2018; Zhang et al., 2018; Konishi et al., 2019; Lu et al., 2019; Xu et al., 2022; Zhang et al., 2024). Functional studies confirmed the crucial role of SWELL1 in cancer progression. In cervical cancer cells, overexpression of *SWELL1* significantly enhances cell migration and invasion (Chen et al., 2023). However, in PAAD cell lines, inhibiting *SWELL1* expression reduces cell migration and invasion ability and significantly reduces the expression of epithelial mesenchymal transition markers and matrix metalloproteinase 2 [MMP2] (Xu et al., 2022). These findings suggest that SWELL1 is closely associated with invasive progression and poor cancer prognosis (Xu et al., 2020; Zhang et al., 2024). However, inhibiting *SWELL1* expression using siRNA technology has minimal effect on HeLa cell proliferation (Sirianant et al., 2016), indicating that the role of SWELL1 varies across different cell types.

SWELL1 regulates the proliferation and migration of cancer cells through multiple signaling pathways (Figure 2). In HCC cells, activation of SWELL1 induces the transition of the cell cycle from the G1 to S phase, thereby promoting cell proliferation (Lu et al., 2019). In-depth research has revealed that SWELL1 activates the cyclin D1/cyclin dependent kinase 2 (CDK2) signaling axis by binding to protein kinase C α (PKCa), while regulating HCC cell migration through the c-Jun N-terminal kinase (JNK) pathway (Lu et al., 2019) (Figure 2). In addition, inhibiting the expression of *SWELL1* in gastric cancer cells not only suppresses cell proliferation and migration (Kurashima et al., 2021), but also leads to changes in the expression of a series of key signaling molecules, including genes related to the p53 signaling pathway (such as *JNK*, *p53*, and *p21*), B cell lymphoma 2 (*Bcl-2*), and Fas cell surface death receptor [*FAS*] (Sørensen et al., 2016; Kurashima et al., 2021) (Figure 2).

In summary, the expression level of SWELL1 is closely related to the invasiveness and progression of cancer, playing a key role in the proliferation and migration of cancer cells by regulating multiple signaling pathways. Although the specific mechanism of action of SWELL1 may vary across distinct cancer types, these findings reveal its important role in the occurrence and development of cancer, providing a theoretical basis for the development of novel anti-cancer treatment strategies targeting SWELL1.

### 4.2 SWELL1 regulates cell death

Apoptosis is a programmed cell death mode strictly regulated by genes that is crucial for tissue homeostasis and overall health (Pedersen et al., 2013; Kumagai et al., 2016). Notably, SWELL1 exhibits a bidirectional regulatory effect that promotes and inhibits apoptosis (Figure 2).

Several studies have confirmed the important role of SWELL1 in promoting apoptosis. When muscle cells are stimulated by apoptosis-inducing factors, the expression of SWELL1 is significantly upregulated (Kenagy et al., 2011). Under oxidative stress conditions, the expression level of SWELL1 is significantly positively correlated with the activation level of the pro-apoptotic transcription factor p53 (Bach et al., 2018; Mao et al., 2023). This transcription factor is an important tumor suppressor that induces apoptosis through various mechanisms, suggesting that SWELL1 promotes apoptosis through a p53-dependent pathway (Figure 2). Rubino et al. validated this hypothesis by using siRNA technology to inhibit the expression of *SWELL1* in malignant glioma cells (Rubino et al., 2018). Their results showed that *SWELL1* silencing significantly reduced cell viability and significantly increased the sensitivity of cells to clinical drugs such as Temozolomide and Carmustine. Mechanistic studies have revealed that inhibiting SWELL1 in glioma cells upregulates the expression of the anti-apoptotic protein Bcl-2 and downregulates the expression of the pro-apoptotic protein Bax, thereby inhibiting the release of cytochrome C and activation of caspase-9/-3, ultimately inhibiting the mitochondria-dependent apoptosis pathway induced by temozolomide (Yang C. et al., 2019) (Figure 2). However, SWELL1 can also exert anti-apoptotic effects under certain conditions. For example, Chen et al. found that NSUN2 mediated 5-methylcytosine (m5C) modification increases *SWELL1* mRNA expression in cervical cancer cells. The modified *SWELL1* mRNA binds more strongly to the RNA-binding protein YBX1, thereby improving RNA stability. This series of changes ultimately activates the PI3K/AKT signaling pathway, inhibits caspase-3 protein expression, and suppresses cell apoptosis (Chen et al., 2023) (Figure 2).

In summary, SWELL1 exhibits complex bidirectional regulatory effects during cell apoptosis (Kenagy et al., 2011; Bach et al., 2018; Rubino et al., 2018; Yang J. et al., 2019), suggesting that its function may be finely regulated by multiple factors. Previous studies have shown that various intracellular and extracellular environmental factors, such as pH, reactive oxygen species (ROS), and ATP, can dynamically regulate the activity and ion permeability of ion channels by affecting their conformation and gating properties (Wang et al., 2017; Chu et al., 2023). Considering that the process of cell apoptosis is often accompanied by significant changes in intracellular pH, ROS levels, and energy status (Ostrovskaya et al., 2014; Liu et al., 2021), whether these factors affect cell apoptosis by regulating the activity of SWELL1 is a question worthy of further exploration.

## 5 Challenges and opportunities in the development of therapeutic drugs using the SWELL1 channel

Previous studies have shown that SWELL1 is closely related to metabolic syndrome, cancer, and various other diseases, highlighting its enormous potential as a drug intervention target; however, the development of therapeutic drugs targeting the SWELL1 channel still faces many challenges (Gunasekar et al., 2022). The primary challenge lies in the mechanism complexity of the SWELL1-LRRC8 complex. As mentioned earlier, SWELL1 has complex interactions and synergistic effects with multiple ion channels and regulatory factors, jointly constructing a precise insulin secretion regulatory network (Best et al., 2011; Stuhlmann et al., 2018). In type 2 diabetes (T2D), pathophysiological factors such as glycolipid toxicity and endoplasmic reticulum stress may further interfere with the assembly and function of the SWELL1-LRRC8 complex (Gunasekar et al., 2022). Therefore, in-depth elucidation of the assembly regulation mechanism of the SWELL1-LRRC8 complex and its molecular mechanism of functional impairment under pathological conditions is key to developing efficient, specific, and safe SWELL1 channel modulators. Secondly, the SWELL1-LRRC8 complex is widely expressed in various tissues and cell types, including pancreatic beta cells, metabolic tissues, and cancer cells (Qiu et al., 2014), which poses a dual challenge of target specificity and safety for drug development. Therefore, an ideal SWELL1 channel modulator should have high tissue selectivity, accurately targeting pancreatic beta cells and insulin sensitive tissues, while minimizing its impact on other tissues and organs, thereby reducing the potential risk of adverse reactions. In addition, given the unclear role of SWELL1’s non-ionic channel function (such as participating in intracellular signal transduction) in metabolic regulation (Zhang et al., 2017; Kumar et al., 2020), its feasibility as a drug intervention target still needs further evaluation.

In addition to its potential in the treatment of metabolic diseases, SWELL1 also has broad application prospects in central nervous system diseases such as neuropathic pain (Yang J. et al., 2019). Research has shown that during peripheral nerve injury, the SWELL1 channel in microglia initiates a series of signaling cascades by releasing ATP, leading to neuropathic pain (Chu et al., 2023). In the small glial cell specific SWELL1 knockout mouse model, neuropathic pain related symptoms were significantly improved, confirming the importance of SWELL1 as a target for neuropathic pain drugs (Chu et al., 2023). In addition, high-throughput drug screening has found that two drugs approved by the Food and Drug Administration (FDA), dicumarol and zafirlukast, can act as VRAC inhibitors and effectively alleviate neuropathic pain (Chu et al., 2023). This discovery provides important clues for the development of novel analgesic drugs targeting SWELL1. Future research can further optimize the pharmaceutical properties of SWELL1 channel drugs; improve their specificity and safety based on these works; explore new drug action sites and lead compounds; and explore the possibility of combining SWELL1 with existing analgesic drugs to achieve synergistic effects, improve efficacy, and reduce adverse reactions.

SWELL1, as a key component of VRAC, not only has ion channel functions to regulate cell volume and ion homeostasis but is also a multifunctional cell membrane protein with non-ionic channel functions such as protein interactions and signal transduction. Therefore, when developing therapeutic drugs targeting SWELL1, it is necessary to comprehensively consider how to effectively regulate both its ion channel function and non-ion channel function. Targeting the SWELL1 ion channel function may have a more direct impact on the physiological state of cells and is expected to improve disease symptoms by regulating cell volume and ion homeostasis. However, due to the widespread expression of VRAC channels in various tissues and cell types, non-selective regulation may cause side effects, thus this strategy may face challenges in specificity and safety. Although targeting the non-ionic channel function of SWELL1 can provide more diverse drug intervention pathways, such as interfering with specific protein interactions or signaling pathways, the mechanism of action may be more complex and requires further research to clarify specific drug targets and regulatory strategies.

## 6 Discussion

In summary, SWELL1 is a multifunctional protein that plays an important regulatory role in metabolic tissues. Its functions are primarily evident in two aspects: on the one hand, SWELL1 is a key component of the VRAC, participating in cell volume regulation by regulating I_Cl(swell)_ current; on the other hand, SWELL1 has an ion channel independent protein function, and can directly participate in metabolic process regulation by interacting with insulin pathway components or lipid metabolism related proteins.

The I_CL(swell)_ current mediated by SWELL1 may be significantly enhanced under cell swelling or ROS stimulation (Matsuda et al., 2010; Zhang et al., 2017). The enhancement of this current may indirectly affect the activity of downstream signaling pathways (such as the PI3K/Akt pathway) by regulating cell volume and membrane potential, thereby participating in metabolic regulation (Wong et al., 2018). Under normal physiological conditions, the activity of I_Cl(swell)_ current is low. Therefore, SWELL1 may primarily and directly regulate metabolic processes through non-channel functions, such as regulating insulin signaling pathways or lipid metabolism related proteins. However, the specific mechanisms of SWELL1 metabolic regulation, particularly the relative contributions and synergistic effects of ion channel and non-channel functions, are still poorly understood. At present, the research on SWELL1 regulating metabolism mainly focuses on metabolic disease models such as obesity or T2D. Under these pathological conditions, The I_Cl(swell)_ current typically exhibits significant activation, which may be due to factors such as obesity related cell swelling, chronic inflammation, and oxidative stress that continuously stimulate the I_Cl(swell)_ current (Matsuda et al., 2010). In this case, inhibiting SWELL1 and I_Cl(swell)_ currents may help improve metabolic disorders. In contrast, under normal physiological conditions with low basal activity of I_Cl(swell)_ current, the effect of SWELL1 inhibition on metabolism may be relatively limited. In addition, under pathological conditions (such as obesity or T2D), the ion channel function and non-channel function of SWELL1 may work together to regulate the metabolic process. For example, the enhancement of I_Cl(swell)_ current may affect metabolic signaling pathways by regulating cell volume and membrane potential; Meanwhile, the non-channel function of SWELL1 may further affect metabolic signaling pathways by directly regulating the activity of related proteins. However, there is currently a lack of research on the quantitative relationship between I_Cl(swell)_ current activity and the severity of metabolic diseases. The systematic evaluation of the correlation between changes in I_Cl(swell)_ current activity and metabolic disorders may help to deepen the understanding of the pathophysiological significance of SWELL1.

In addition to participating in metabolic regulation, SWELL1 may also serve as an important bridge between metabolic abnormalities and pathological processes such as inflammation (Hua et al., 2020). Previous studies have shown that SWELL1 can participate in the inflammatory response of metabolic diseases by regulating the activation of NLRP3 inflammasomes (Catalán et al., 2024). This suggests that SWELL1 may play an important role in the inflammatory response caused by metabolic abnormalities, and its specific molecular mechanism deserves further exploration. In addition, the role of SWELL1 in skeletal muscle metabolism regulation and exercise adaptation is gradually being revealed. In particular, SWELL1 deficiency can lead to skeletal muscle atrophy and decreased exercise endurance (Kumar et al., 2020), suggesting that SWELL1 may affect the body’s exercise performance and metabolic status by regulating the structure and function of skeletal muscles. In depth research on the regulatory mechanism of SWELL1 on skeletal muscle metabolism and exercise adaptation is expected to provide new ideas for elucidating the molecular basis of exercise prevention and treatment of metabolic diseases. However, the specific mechanism of action of SWELL1 in skeletal muscle is still poorly understood. Further exploration and clarification are needed on how its expression and activity respond to exercise stimuli, how it regulates adaptive changes in skeletal muscle, and ultimately affects systemic metabolism.

Given the crucial role of SWELL1 in the occurrence and development of metabolic diseases, and its regulatory potential in multiple aspects such as the insulin pathway, inflammatory response, and skeletal muscle metabolism, future research should focus on the following aspects: (1) elucidating the molecular links between SWELL1 and metabolic disorders, such as insulin resistance, to uncover its role in maintaining systemic metabolic homeostasis; (2) exploring how SWELL1 regulates skeletal muscle structure and function, and its potential to enhance metabolism through exercise; (3) investigating the interactions between SWELL1 and pathological processes like inflammation and cellular stress to deepen understanding of metabolic disease pathogenesis; and (4) developing novel drugs targeting SWELL1 to treat metabolic diseases, thereby laying the groundwork for the next-generation of anti-diabetic and anti-obesity therapies.

In conclusion, research on SWELL1’s biological functions has made notable strides, highlighting its vast potential for application. Future efforts are expected to unravel the comprehensive mechanisms of SWELL1, identify early biomarkers, and establish innovative treatment strategies for metabolic diseases. This progress will pave the way for precise prevention and management of conditions such as diabetes and obesity.
